# Acceptability of a text message-based intervention for obesity prevention in infants from Hawai‘i and Puerto Rico WIC

**DOI:** 10.1186/s12884-019-2446-9

**Published:** 2019-08-13

**Authors:** Cheryl L. K. Gibby, Cristina Palacios, Maribel Campos, Rafael E. Graulau, Jinan Banna

**Affiliations:** 1 Department of Human Nutrition, Food and Animal Sciences, College of Tropical Agriculture and Human Resources, University of Hawai‘i at Mānoa, Agricultural Sciences 216, 1955 East-West Rd, Honolulu, HI 96822 USA; 2 Dietetics and Nutrition Department, Robert Stempel College of Public Health & Social Work, Florida International University, 11200 SW 8th Street, AHC 5–313, Miami, FL 33199 USA; 3 Dental and Craniofacial Genomics Unit, Endowed Health Services Research Center, Medical Sciences Campus, University of Puerto Rico, PO Box 365067, San Juan, PR 00936-5067 USA; 4 Nutrition Program, Graduate School of Public Health, Medical Sciences Campus, University of Puerto Rico, PO Box 365067, San Juan, PR 00936-5067 USA

**Keywords:** Text message, Low income, Childhood obesity, Infants, WIC, Breastfeeding, Complementary feeding, Randomized controlled trial, Mobile health, Minority

## Abstract

**Background:**

Low-income and minority children are at increased risk for obesity. Text messaging offers advantages for delivering education, but few studies have assessed the acceptability of text messaging in interventions aimed at preventing excessive weight gain in infants. This study investigated the acceptability of a text message-based intervention for prevention of excessive weight gain in infants from Hawai‘i and Puerto Rico WIC clinics.

**Methods:**

The four-month text message based intervention designed to improve infant feeding practices and reduce excessive weight gain was a randomized controlled trial that included mothers with infants ages 0–2 months at baseline. Participants in the intervention arm received 18 text messages (1/week) promoting breastfeeding and appropriate complementary feeding. Acceptability of the intervention was assessed from participant retention, satisfaction, and evidence of behavior change in a sequential multimethod approach, quantitatively from questions sent via text and qualitatively during the in-person exit interview. The final analysis included 80 mother-infant pairs from the intervention arm.

**Results:**

When asked about messages liked and disliked the most, the majority of responses via text indicated that they liked all messages. From the qualitative analyses, most participants reported that all messages were useful and that the messages led them to make changes in the way they fed their infants. Participant retention was good at 78.4%.

**Conclusions:**

The intervention was acceptable to participants based upon participant retention, measures of satisfaction, and reports of behavior change. Results may inform development of mobile health programs for minority childhood obesity prevention.

**Trial registration:**

ClinicalTrials.gov Identifier; NCT02903186; September 16, 2016.

## Background

Childhood obesity is a serious public health problem which increases the risk of developing other chronic disease later in life, such as diabetes, cancer and cardiovascular disease [1]. The comorbidities associated with childhood obesity affect almost every system in the body, including the endocrine, gastrointestinal, pulmonary, cardiovascular and musculoskeletal systems [1]. Low-income and minority children are especially at risk for obesity [2], with Hispanic children having increased odds of rapid infancy weight gain [3] and Native Hawaiian or other Pacific Islander (NHOPI) children having higher weight early in life [4]. In the US, 40% of low-income one-year-olds and 30% of two to five-year-olds participating in the Special Supplemental Nutrition Program for Women, Infants, and Children (WIC) were overweight or obese in 2014 [5, 6]. Early intervention to support optimal weight gain, therefore, may be especially beneficial in these groups.

Breastfeeding is protective against obesity [7, 8], with more protection conferred as exclusivity and duration of breastfeeding increases [9]. Early discontinuation of breastfeeding and early introduction of solid foods is associated with excessive weight gain [3, 10]. Currently, the US breastfeeding rate at six months is below the Healthy People 2020 goal of 60.6% [11], and in some locations the rate is especially low, such as in Puerto Rico at 38.1% [12]. There is a continued need to promote and support appropriate breastfeeding practices.

Infant health studies using text messaging to deliver infant feeding information have reported significantly lower prevalence of overweight and obesity in infants at six months old [13] and improved exclusive breastfeeding duration [14]. Other studies have used text messaging to improve infant weight [15] and to improve adherence to the infant immunization schedule [16]. However, there have not been many text message-based studies aimed at preventing childhood obesity.

Text message-based interventions for health promotion and disease prevention deliver educational information, reminders, questions, tips, and other correspondence via text messages. Mobile phones are useful tools for health interventions because of their increasing popularity, ease of usability, and people’s tendency to always carry them [17]. Mobile health (mHealth) programs are potentially more effective than traditional face-to-face interventions, which are more labor intensive, time consuming, and expensive [18]. Minority groups, those with lower household income, and those with lower levels of completed education were reported to text more than other groups [19], indicating that text message-based interventions may be most effective in these groups.

Mobile technology may also help to increase participant retention in studies. For example, a health behavior study reported that children in the text message group had better participant retention (72%) in comparison to the non-technology (39%) and control (50%) groups, and significantly greater adherence (43%) to self-monitoring in comparison to the non-technology (19%) groups [20]. Some nutrition studies utilizing text messaging have reported excellent participant retention rates ranging from 80 to 97% [21–23]. Participant retention has been commonly used in studies to assess acceptability [24].

Assessing acceptability is a necessary component in determining the effectiveness of an intervention [24]. For example, a study on acceptability and program development for a mobile phone depression prevention intervention for adolescents reported findings regarding participation rates, how well participants liked the messages, usefulness of the messages, and aspects of behavioral change [25]. Text message-based interventions have been shown to be well accepted in many nutrition studies [20, 26–28]; however, not many infant health studies have assessed acceptability of the interventions. Assessing acceptability of text message-based interventions contributes to the improvement of mHealth intervention designs.

The objective of this study was to determine the acceptability of a text message-based intervention for obesity prevention in infants from lower socioeconomic backgrounds in Hawai‘i and Puerto Rico using qualitative and quantitative methods to assess participant retention, satisfaction, and evidence of behavior change. Major themes regarding the usefulness of the text messages and how participants were influenced to change behaviors were investigated, and messages liked the most and least were determined.

## Methods

### Participants

The four-month text message-based intervention was designed to improve infant feeding practices and reduce excessive infant weight gain. Mother-infant pairs in WIC in Hawai‘i and Puerto Rico were recruited. Infants were 0–2 months at baseline. Eligibility criteria for mothers/caregivers included the following: at least 18 years old, owned a mobile phone with unrestricted texting capabilities, responsible for caring for the infant, willing to complete the entire study, and able to read. Additionally, inclusion criteria required the infant to have been no more than two months old at baseline, to have been born after 37 weeks of gestation, to be on a normal diet and free from disabilities that hinder movement, and to have had birthweight at or between the 10th and 90th percentiles as indicated by the World Health Organization (WHO) growth charts [29]. Study procedures were approved by the institutional review boards at the University of Hawai‘i at Mānoa and the University of Puerto Rico, Medical Sciences Campus. Written informed consent was obtained prior to data collection. Additional details about methods and outcomes for the trial have been published elsewhere [30, 31].

### Intervention design

To send and receive messages, a third-party web-based text messaging platform was used as the Internet gateway. We ensured consistency in the messages as they were developed in culturally appropriate English and Spanish, for the selected target populations. Messages in Hawai‘i were sent in English and messages in Puerto Rico were delivered in Spanish. Prior to the study, some of the messages were pre-tested with five women in Puerto Rico, who all reported that the utility and frequency of messages were excellent [32]. Details regarding intervention design have been published elsewhere [31].

### Intervention implementation

Participants’ cell phones were registered in the web-based text messaging platform at baseline.

Over the course of four months, participants received 18 text messages sent on varying days and times, at a frequency of one message per week. Participants were informed they could send comments or questions back if desired. Approximately the first half of the intervention group messages focused on reinforcing WIC breastfeeding messages, with the remainder of messages focused on complementary feeding practices such as preventing overfeeding, delaying introduction of solid foods, and reducing juice consumption. Information on the control messages has been published previously [31].

Follow-up visits were conducted in person four months after the participant’s baseline visit [31]. Measures from the baseline visit were repeated and participants in the intervention group completed an interview during which qualitative data regarding acceptability of the study was collected.

### Satisfaction

During the intervention but separate from the main text messages, participants in the intervention arm were sent seven short quantitative and qualitative questions approximately every other week, starting after the second week, and were told that these text messages required a response. Two questions, “Which text message did you like the most so far?” and “Which text message did you like the least so far?” were analyzed. Responses to the other quantitative questions were reported elsewhere [30].

### Evidence of behavior change

At the follow-up visit, they also completed a qualitative interview regarding helpfulness of the messages, problems with receiving messages, ways in which receiving the messages influenced or changed feeding practices, and overall feelings about receiving the messages. Interviewers were trained in techniques and protocol by the principal investigators. Responses were handwritten by the interviewers and later transcribed. Participants were asked six open-ended questions and were encouraged to elaborate on answers. Responses to the following three qualitative questions were analyzed: “Thinking back on the messages you were sent, which text messages were the most useful to you in feeding your infant? Why?”, “Thinking back on the messages you were sent, which text messages were the least useful to you in feeding your infant? Why”, and “Were there any messages that led you to feed your baby in a certain way, or make changes in what you might normally do? If so, which ones (text messages) were these, and how did they influence the way you feed your baby?” Responses to the other three qualitative questions are reported elsewhere [30].

### Participant retention

Eighty participants from the intervention group completed the follow-up visit and qualitative interview. Twenty-two participants from the intervention group were lost to follow up: Hawai‘i (*n* = 15) and Puerto Rico (*n* = 7). Details explaining reasons participants were lost to follow-up were published previously [29].

### Data analyses

For baseline characteristics, descriptive statistics were presented using frequencies and percentages, or using means and standard deviations. Chi-squares tests, Fisher’s exact tests, and two-sample t-tests were conducted to investigate the differences in characteristics between the final analysis group and the group that was lost to follow-up. For all analyses, a *p*-value of < 0.05 was considered statistically significant. Analyses were performed using SAS University Edition (SAS Institute Inc., Cary, NC).

Content analysis was used to analyze qualitative data and report on themes from participant responses. Transcribed interviews were entered into NVivo Pro for Windows (QSR International, Inc., Burlington, MA) by two coders, one in Hawai‘i and one in Puerto Rico. A shared codebook was developed prior to coding and was updated during coding. Inter-rater reliability was tested prior to coding using four transcripts until a kappa value of 0.95 was achieved between coders [33]. Each coder completed coding for all transcripts at his/her site. Then, coders independently identified themes by examining frequencies of codes. Via conference call, results were compared and discussed until agreement was reached.

## Results

Thirty-seven participants (46.3%) from Hawai‘i and 43 participants (53.8%) from Puerto Rico were included in the final analysis. Characteristics of the intervention group, final analysis group, and group that was lost to follow-up are shown in Table 1. Comparing participants in the final analysis group (*n* = 80) with those who did not complete the study (*n* = 22), no statistically significant differences were found for site, pregravid body mass index (BMI), education, race/ethnicity (other than White), parity, pregnancy complications (such as diabetes, hypertension, or anemia), infant gender, being up-to-date with vaccines, taking vitamins while breastfeeding, maternal age (as a continuous variable), gestational age at birth, or gestational weight gain. Women who were White (*p* = 0.04) were less likely to have been lost to follow-up. In comparison to women in age group 25–31 years, women aged 18–24 years and women aged 32 years and older were more likely to be lost to follow-up (*p* = 0.02).

**Table 1:** Distribution of select maternal and infant characteristics, n (%), for the groups in the text message-based intervention acceptability study

	Total intervention (*n* = 102)	Final analysis (*n* = 80)	Lost to follow-up (*n* = 22)	*p*-value
Site				0.068^a^
Hawai‘i	52 (51.0)	37 (46.3)	15 (68.2)	
Puerto Rico	50 (49.0)	43 (53.8)	7 (31.8)	
*Maternal factors*				
Pregravid BMI (mean [SD])	26.6 [6.9]	26.7 [7.1]	26.4 [6.0]	0.873^d^
Age (mean [SD])	26.9 [5.3]	27.0 [5.0]	26.5 [6.4]	0.688^d^
Age group				0.024^b^*
18–24 years	42 (41.2)	30 (37.5)	12 (54.6)	
25–31 years	38 (37.3)	35 (43.8)	3 (13.6)	
32–39 years	22 (21.6)	15 (18.8)	7 (31.8)	
Race^c^/ethnicity				
Hispanic	62 (62.0)	52 (65.0)	10 (50.0)	0.216^a^
Native Hawaiian or Other Pacific Islander	22 (21.6)	17 (21.3)	5 (22.7)	0.881^a^
Asian	22 (21.6)	16 (20.0)	6 (27.3)	0.463^a^
American Indian or Alaska Native	5 (4.9)	5 (6.3)	0 (0)	0.582^b^
Black or African American	15 (14.7)	14 (17.5)	1 (4.6)	0.181^b^
White	34 (33.3)	31 (38.8)	3 (13.6)	0.039^b^*
Education				0.449^b^
Less than college	49 (49.5)	38 (47.5)	11 (57.9)	
Some college	22 (22.2)	20 (25.0)	2 (10.5)	
College degree or higher	28 (28.3)	22 (27.5)	6 (31.6)	
Parity				0.126^b^
1	41 (40.2)	28 (35.0)	13 (59.1)	
2	36 (35.3)	32 (40.0)	4 (18.2)	
3	15 (14.7)	11 (13.8)	4 (18.2)	
4 or more	10 (9.8)	9 (11.3)	1 (4.6)	
Use of prenatal vitamins	97 (95.1)	78 (97.5)	19 (86.4)	0.066^b^
Pregnancy complications	43 (42.2)	32 (40.0)	11 (50.0)	0.400^a^
Took vitamins while breastfeeding	58 (56.9)	47 (58.8)	11 (50.0)	0.463^a^
Gestational age (weeks; mean [SD])	39.0 [1.1]	39.1 [1.1]	38.8 [1.1]	0.426^d^
Gestational weight gain (lb; mean [SD])	27.7 [11.2]	27.6 [11.1]	28.5 [12.1]	0.749^d^
*Infant factors*				
Male	51 (50.0)	39 (48.8)	12 (54.6)	0.630^a^
Female	51 (50.0)	41 (51.3)	10 (45.5)	
Up-to-date with vaccinations	87 (85.3)	68 (85.0)	19 (86.4)	1.00^b^

Note: Column percentages; p-value represents final analysis vs lost to follow-up;

*p < 0.05; ^a^Analysis by Chi-square test; ^b^Analysis by Fisher’s exact test; ^c^Includes all races for mixed participants; ^d^Analysis by t-test, pooled

### Participant retention

Participant retention was 78.4%. In Hawai‘i, the rate was 71.2%, and in Puerto Rico, the rate was 86%.

### Satisfaction

Results from text messaged questions regarding which messages participants liked the most and the least are reported in Table 2 and Table 3, respectively. Participants responded via text message, and most indicated that they had no preference in topic as they liked all messages the most (*n* = 8). Thereafter, participants in Hawai‘i most enjoyed the text messages about offering breastmilk in a bottle or cup (*n* = 2) and breastfeeding often during growth spurts (n = 2), while participants in Puerto Rico most enjoyed the educational information about starting (*n* = 7) and implementing (*n* = 4) solid food feeding. Most respondents indicated that they liked all messages when asked about which message they liked the least (*n* = 12).

**Table 2:** Responses from participants in week 14 regarding which message they liked the most in the text message-based intervention

Week delivered	Message	Number of participants: Puerto Rico (*n* = 15)	Number of participants: Hawai‘i (*n* = 17)
2	When breastfeeding, make sure the nipple and the area around is inside baby’s mouth. If baby eats from the tips, they will crack. Always correct the position.	1	
3	Breastfeeding is the best way to feed your baby, but it may be hard. Put your baby to your breast and you will have more milk. Ask for help.		1
6	Breastfeed your baby from the same breast until it feels empty. That way, your baby gets the fat that comes at the end and will be full longer.		1
8	Your milk is the best food for baby for the first 6 months of life. If you cannot put your baby directly to your breast, you can give it in a bottle or cup.		2
8	You can tell if you have enough milk by counting wet diapers. Your baby should have 6 or more wet diapers every day after the 4th day of birth.		1
9	Babies have growth spurts and want to breastfeed often, which increases hunger. Your baby will drink more to increase your milk for 3 days. This is normal.		2
10	If you give milk in a bottle, do not add other foods such as baby cereal or baby food. If your baby seems full, do not force him/her to finish it.	1	1
13	Prepare your milk stock by extracting milk at the end of every feeding and put it in the fridge in a clean bottle. At the end of the day you will have 2–3 oz.	1	1
14	Do not put your baby to sleep with the bottle or cup. The milk residue can lead to cavities and to excess weight.		1
15	Your baby is ready to eat when he/she sits on his/her own, opens his/her mouth, chews and leans toward foods. Wait until 6 months to start feeding other foods.	7	
16	When your baby is 6 months, you can give meat, cereals with iron, or vegetables (puree), 1 at a time and using a spoon. Wait 3 days before giving a new food.	4	
NA	All messages	1	7

Note: *NA* Not applicable

**Table 3:** Responses from participants in week 16 regarding which message they liked the least in the text message-based intervention

Week delivered	Message	Number of participants: Puerto Rico (*n* = 9)	Number of participants: Hawai‘i (*n* = 11)
3	To start breastfeeding again, ask for help. You only can give your milk if you put your baby to your breast often to make milk.	1	
7	While breastfeeding, you do not need to eat a special diet or beverage, you only need to be hydrated. Drink 8–10 glasses of water every day.		1
11	Feed your baby when he/she moves his/her lips, sucks his/her hands and turns his/her head searching for the breast. Crying does not always mean hunger.	1	
12	If you need to work or study, extract milk every 2–3 h to keep up your milk production. There are laws that protect you to do this.		1
13	Prepare your milk stock by extracting milk at the end of every feeding and put it in the fridge in a clean bottle. At the end of the day you will have 2–3 oz.	1	
15	Your baby is ready to eat when he/she sits on his/her own, opens his/her mouth, chews and leans toward foods. Wait until 6 months to start feeding solid foods.	1	1
18	Baby juice has sugar that babies do not need. Instead of juice, give water or fruits pureed or blended with water. This will help your baby stay healthy.	1	
NA	Like all messages	4	8

Note: *NA* Not applicable

Results from in-person interview questions on acceptability with exemplifying quotations are presented in Tables 4, 5 and 6. Themes are reported in order of prominence, according to coding frequencies. Prevalent themes from the qualitative data supported the outcomes of the quantitative observations indicating that the messages were useful and well-liked.

**Table 4:** Coding frequencies for most prevalent themes and exemplifying quotations regarding which text messages participants (n = 80) felt were the most useful in feeding their infant

Prevalent themes and coding frequencies	Explanation of theme	Exemplifying quotation
Most-feeding knowledge (HI = 9, PR = 6)	Messages informed or reminded participant of feeding facts and tips.	“Choosing formula with iron was helpful because I didn’t really know much about iron.”
Most-breastfeeding knowledge (HI = 3, PR = 12)	Messages informed or reminded participant of breastfeeding facts and tips.	“Now I have an 8-year-old son and these are things (about breastfeeding) I never knew.”
Most-all (HI = 6, PR = 8)	All messages were found useful.	“Yes, all the information was useful and I replied back to some of them.”
Most-breastfeeding technique (HI = 7, PR = 7)	Participant gained knowledge of techniques such as proper positioning, pain management, and milk production.	“I always had a hard time breastfeeding, especially getting baby latched on. So this message was a good reminder for latching.”
Most-breastfeeding signs (HI = 7, PR = 3)	Messages informed participant of hunger signs that indicate when to breastfeed.	“I thought in the beginning that crying meant he was hungry, so we wasted a lot of milk trying to feed him when he wasn’t hungry.”
Most-feeding application (HI = 8, PR = 1)	Messages helped participant apply proper feeding techniques and information, such as counting diapers and offering foods with a spoon.	“I used spoon to feed and watched baby’s behavior to new foods like when she goes to rice cereal and opens her mouth.”

Note: *HI* Hawai‘i, *PR* Puerto Rico

**Table 5:** Coding frequencies for most prevalent themes and exemplifying quotations regarding which messages participants (n = 80) felt were the least useful in feeding their infant

Prevalent themes and coding frequencies	Explanation of theme	Exemplifying quotation
Least-none (HI = 25, PR = 18)	All messages were found useful.	“All were useful in one way or another.”
Least-breastfeeding not applicable (HI = 5, PR = 7)	Breastfeeding information did not apply to the participant.	“Had to stop breastfeeding before 6 months due to MD’s order, this message didn’t apply.”
Least-breastfeeding known (HI = 3, PR = 7)	Participants already knew the breastfeeding information.	“I already knew.”

Note: *HI* Hawai‘i, *PR* Puerto Rico

**Table 6:** Coding frequencies for most prevalent themes and exemplifying quotations regarding how messages changed the way participants (n = 80) fed their infant

Prevalent themes and coding frequencies	Explanation of theme	Exemplifying quotation
Changes-none none (HI = 7, PR = 8)	Participant made no changes.	“No, did not change the way I fed my baby.”
Changes-breastfeeding knowledge (HI = 2, PR = 10)	Messages led participant to apply correct breastfeeding techniques.	“It helped me produce more breast milk with the correct technique.”
Changes-feeding talk gestures (HI = 6, PR = 3)	Participant started talking to baby during feedings and learned to observe baby’s gestures to indicate hunger.	“Knowing crying doesn’t always mean hunger helped me pay more attention to baby’s cues.”
Changes-feeding solid time (HI = 3, PR = 6)	Participant decided to wait for suggested time to start feeding infant solid foods.	“Although some people told me to feed solid food to baby, I waited until 5–6 months.”
Changes-none already knew (HI = 6, PR = 3)	Participant did not make any changes because they already knew the information.	“No, I already knew the information in the messages.”
Changes-feeding caries (HI = 5, PR = 3)	Participant stopped putting her infant to sleep with a bottle.	“The message about putting baby to sleep with a bottle- I was doing this and then stopped doing it after reading the message.”

Note: *HI* Hawai‘i, *PR* Puerto Rico

### Evidence of behavior change

Participants expressed that messages that delivered previously unknown information or that were remindful were the most useful in feeding their infants (*n* = 15). Thereafter, participants indicated that all messages (*n* = 14) and messages about breastfeeding techniques (n = 14) were most useful for feeding.

Regarding which messages were the least useful for feeding, the majority of participants reported that all messages were useful (*n* = 43). Other responses indicated that breastfeeding messages were not useful to participants who were not breastfeeding (*n* = 12) and that breastfeeding messages were not useful if participants already knew the information (*n* = 10).

Most participants reported that the messages led them to make changes in the way they fed their infants, such as by applying correct breastfeeding techniques (n = 12), talking to their infant during feedings and observing hunger cues (*n* = 9), and waiting to start solid foods (n = 9). However, some participants reported that they made no changes in feeding (*n* = 24).

## Discussion

The intervention acceptability was very good, as indicated by high participant retention, a high rate of liking messages, reports of finding all messages useful in feeding infants and success of messages in changing behaviors.

Sekhon et al. proposed that acceptability is based on emotional and cognitive responses to the intervention and could be assessed prior to or after the intervention [24]. Furthermore, reviews of studies have reported that participant retention or satisfaction measures are often used as proxies for acceptability [24, 34, 35]. Assessed at the end of the intervention, acceptability, as indicated by participant retention (78.4%), satisfaction, and evidence of behavior change, was good in the current study. Satisfaction was evidenced by most participants liking all the messages and finding all messages useful in feeding their infants. Participants also indicated that the messages were successful in changing behaviors as most participants (*n* = 56) stated that they adjusted their feeding practices. This finding is in line with other text message-based intervention studies that have reported text messaging is effective in behavioral change [26, 36–38].

According to the theoretical framework of acceptability (TFA), acceptability is represented by seven factors: participants’ feelings about the intervention (affective attitude); the amount of effort required to participate (burden); ethicality; extent that benefits, profits, or values are sacrificed to participate (opportunity cost); perceived effectiveness; self-efficacy; and intervention coherence [24]. Based upon this definition of acceptability, the current study has investigated the construct of affective attitude in responses about liking the messages and the construct of perceived effectiveness in behavioral changes that were made by participants in response to messages. Evidence of acceptability for this intervention which support other constructs of the TFA were reported previously: convenience was cited most as what participants liked about the intervention (burden); most participants reported no problems in participating (self-efficacy and intervention coherence); and participants most frequently reported enjoying the experience (ethicality) [30]. Therefore, the current study offers further evidence of the acceptability of the intervention and presents insight into which messages were most and least useful in feeding.

Improvements could be made to the intervention. For example, future studies may consider only delivering breastfeeding messages to the formula-feeding group during the first several weeks since they were second most cited as the least useful messages for those who were not breastfeeding. Instead, more messages pertaining to formula and solid food feeding may be more useful in this intervention arm. Also, although no participants during the current study restarted breastfeeding, it may be beneficial in future studies to have another set of messages specific for the needs of relactating mothers who decide to start breastfeeding after reading the text messages. At the baseline visit, mothers in the formula group could be informed that they can notify researchers by texting if they start breastfeeding. At that time, mothers would receive messages specific to relactation since their needs differ from women who have not had a break in lactation [39].

As markers of acceptability, satisfaction measures and participant retention may be confounded by other factors, such as incentives or accessibility of the intervention site [24, 34]. Therefore, it may be important to also assess anticipated acceptability prior to the intervention, which would allow researchers to modify aspects of the intervention for greater acceptability and participation [24]. Moreover, conducting assessments before and after the intervention allows for a more comprehensive view of acceptability.

### Limitations of the study

The current study has several limitations. First, while acceptability of the intervention was assessed, the results reported in this publication do not indicate whether or not the intervention does in fact lead to changes in behavior or weight. The outcomes of the trial have been reported in a separate publication. In addition, due to the capabilities of the web-based text messaging platform, once a text message question was delivered, participants were allowed to respond for the following 12 h only. After the 12-h window, the server did not record responses. The number of responses to the two text questions investigated in this study was low (HI: 37.8%; PR: 27.9%) but it is possible that additional delayed responses were not recorded by the server. Similarly, some messages were categorized by the server as “sent-awaiting confirmation” as some phone carriers did not allow delivery status to be known. In these cases, to ensure that participants were receiving the messages, research staff monitored participants’ activity and contacted them if “sent-awaiting confirmation” appeared repeatedly. Messages that were categorized as “bounced” were resent and the participant was contacted if bounced messages occurred repeatedly. In Puerto Rico, a problem requiring an additional server for two popular phone carriers caused four participants to receive only 28–67% of messages, as reported previously [31]. Finally, some participants (*n* = 26) were not reachable for the follow-up visit despite efforts to contact them by phone call, text message, email, or voicemail.

## Conclusion

Reflected by participant retention, measures of satisfaction, and reports of behavior change, the text message-based intervention was found to be acceptable. These results may be used in the further development of text message-based interventions and may inform strategies for childhood obesity prevention. For example, these results could contribute to the development of new mHealth programs at WIC clinics aimed at educating mothers about breastfeeding and other feeding practices. Further research is needed to determine whether such programs may translate into a population-based reduction in childhood obesity.

## Data Availability

Data available from the corresponding author upon request (with names redacted).

## Abbreviations

BMI: Body mass index
mHealth: Mobile health
NHOPI: Native Hawaiian or other Pacific Islander
TFA: Theoretical framework of acceptability
WHO: World Health Organization
WIC: Special Supplemental Nutrition Program for Women, Infants, and Children
